# Usefulness of a Tailored eHealth Service for Informal Caregivers and Professionals in the Dementia Treatment and Care Setting: The eHealthMonitor Dementia Portal

**DOI:** 10.2196/resprot.4354

**Published:** 2016-04-05

**Authors:** Sandra Schaller, Velislava Marinova-Schmidt, Manuela Setzer, Haridimos Kondylakis, Lena Griebel, Martin Sedlmayr, Elmar Graessel, Juan Manuel Maler, Stefan Kirn, Peter L Kolominsky-Rabas

**Affiliations:** ^1^ Interdisciplinary Centre for Health Technology Assessment (HTA) and Public Health, Friedrich-Alexander-University of Erlangen-Nürnberg Erlangen Germany; ^2^ Institute of Computer Science, Foundation for Research and Technology - Hellas (FORTH) Heraklion Greece; ^3^ Chair of Medical Informatics Friedrich-Alexander-University of Erlangen-Nürnberg Erlangen Germany; ^4^ Centre for Health Services Research in Medicine, Department of Psychiatry and Psychotherapy, University Hospital Erlangen, Friedrich-Alexander-University of Erlangen-Nürnberg Erlangen Germany; ^5^ Department of Psychiatry and Psychotherapy, Friedrich-Alexander-University of Erlangen-Nürnberg Erlangen Germany; ^6^ Institut für Health Care & Public Management, Information Systems 2, University of Hohenheim Hohenheim Germany

**Keywords:** eHealth, web portal, decision aid, personalized support, dementia, Alzheimer’s disease, informal caregiver, medical professional

## Abstract

**Background:**

The European eHealthMonitor project (eHM) developed a user-sensitive and interactive Web portal for the dementia care setting called the eHM Dementia Portal (eHM-DP). It aims to provide targeted support for informal caregivers of persons with dementia and professionals.

**Objective:**

The objective of this study was to assess the usefulness and impact of the eHM-DP service in the dementia care setting from two user perspectives: informal caregivers and professionals.

**Methods:**

The evaluation study was conducted from June to September 2014 and followed a before-after, user-participatory, mixed-method design with questionnaires and interviews. The used intervention was the eHM-DP: an interactive Web portal for informal caregivers and professionals that was tested for a 12-week period. Primary outcomes for caregivers included empowerment, quality of life, caregiver burden, decision aid, as well as perceived usefulness and benefits of the eHM-DP. Primary outcomes for professionals involved decision aid, perceived usefulness, and benefits of the eHM-DP.

**Results:**

A total of 25 informal caregivers and 6 professionals used the eHM-DP over the 12-week study period. Both professionals and informal caregivers indicated perceived benefits and support by the eHM-DP. In total, 65% (16/25) of informal caregivers would use the eHM-DP if they had access to it. Major perceived benefits were individualized information acquisition, improved interaction between informal caregivers and professionals, access to support from home, and empowerment in health-related decisions (PrepDM Score: 67.9). Professionals highlighted the improved treatment and care over the disease course (83%, 5/6) and improved health care access for people living in rural areas (67%, 4/6). However, there was no improvement in caregiver burden (Burden Scale for Family Caregivers) and quality of life (EuroQol-5D-5L) over the study period.

**Conclusions:**

Our study provides insight into the different user perspectives on an eHealth support service in the dementia treatment and care setting. These results are of importance for future developments and the uptake of eHealth solutions in the dementia domain and reinforce the importance of early user involvement. Turning to the primary target of the eHM-DP service, our findings suggest that the eHM-DP service proved to be a valuable post-diagnostic support service, in particular for the home-based care setting. Further research on a larger scale is needed to enhance the implementation in existing health care infrastructures.

##  Introduction

 Planning for the treatment and care provision of increasing numbers of persons with dementia (PwD) and their informal caregivers has become an urgent global health service task. Need for care in dementia starts early and increases with disease severity, affecting multiple dimensions such as medical treatment and care, support for household, financial, and social activities, up to almost constant supervision in the severe stage. Thus, dementia has a high impact on PwDs, families (informal caregivers), and health care systems [1]. On average, 70% of persons with dementia are cared for at home. In particular, spouses and children provide extensive care and communicate with professionals, while facing many challenges. They often suffer from higher physical and emotional burden [1-7] in contrast to informal caregivers caring for a person without dementia [8]. In addition, the personal burden of informal care is one of the main reasons for nursing home transfers [9,10]. Given that there is currently no cure for dementia, care concepts and services that provide assistance for persons with dementia are essential. Although several support services exist, they are highly underutilized. Brodaty et al [11] identified the following four major reasons for non-use of support services: caregivers do not perceive the need of the services, reluctance to use the services, the service characteristics, and the lack of information regarding the availability of support services. A recent review highlights the importance of tailoring support services to the needs of caregivers as well as improving access to services [12]. Against this background and due to the projected increase in the number of PwD worldwide [13], there is an urgent need for cost-effective support services for informal caregivers. In this context, several studies highlight the potential of eHealth services, due to the increasing availability of Internet access and the benefit of flexibility, facilitated accessibility, and personalization of the service [14-19].

Therefore, the European eHealthMonitor project (eHM) developed a user-sensitive and interactive Web portal for dementia care: the eHM Dementia Portal (eHM-DP). By providing information and access for local support services from home and information that is tailored to the needs of the users, the eHM-DP aims to increase the use of support services. It aims to provide targeted and personalized support for both informal caregivers of persons with dementia in a home-based care setting and professionals [20]. Especially during the course of the disease, medical professionals play an important role with regard to treatment and care for the PwD and communication with informal caregivers is crucial in order to provide the appropriate treatment and care.

Currently, the majority of Internet-based, supportive interventions for informal caregivers in dementia are websites or specific educational programs. A recent review identified six (of 14) interventions that included a professional [21]. However, only a minority of studies that were identified in two recent reviews [21,22] were similar to the eHM-DP with respect to provision of context-sensitive information [23,24] or interaction functionalities [25-27]. In comparison, the eHM-DP is unique by combining individualized (need-tailored) information and interaction functionalities for both informal caregivers and professionals. Overall, the eHM-DP differs from previous eHealth service solutions for informal caregivers of PwD by a combination of seven major aspects, which were identified based on current reviews [21,22] and the involvement of users in the initial development of the program: (1) interactive and personalized portal with own account, (2) computerized communication between professionals and informal caregivers, (3) tailored support services according to user-specific entries in caregiving diaries, (4) focus on caregiver empowerment and decision aid, (5) the perspectives of medical professionals, (6) provision of individual and longitudinal data about the home-based care setting and course of the disease of the PwD (ie, symptoms, medication, well-being), and (7) provision of individual and longitudinal data of caregiving tasks and caregiver burden. A further advantage of the eHM-DP is the professionals’ access to information from health care parameters of PwDs and caregiver burden of informal caregivers.

The purpose of this study was to assess the usefulness and impact of the eHM-DP. The focus was on perceived usefulness that was assessed by the attitude toward using, perceived benefits, concerns, and recommendations of informal caregivers and professionals. In addition, the impact on informal caregivers (quality of life, caregiver burden) was explored.

## Methods

### Description of the eHM Dementia Portal

We designed a Personal eHealth Knowledge Space (PeKS) as an aggregation of all knowledge sources relevant for the provision of individualized personal eHealth services, featuring individualized support and a personalized Web portal that enables interactions with professionals.

Findings of a recent review from Boots et al [22] indicated that multicomponent interventions, combining tailored information with interaction, are the most promising. The technical design of the eHM-DP was realized by service-oriented architecture based on the open source Web platform, Liferay; modeling and semantic knowledge engineering methods; and multiagent systems (MAS) [28,29]. Based on the aforementioned technologies and a rapid and iterative design process between technical and medical partners, the caregivers’ needs were integrated based on (1) a caregiver focus group, (2) interviews with three medical professional experts in the field, and (3) reviews of current scientific literature [2,3,5,21,22,30-33]. Based on these findings, we decided to involve two important professional groups within the eHM-DP: medical and social professionals. Whereas medical professionals are important for medical treatment and care for the PwD (focus on PwD), social professionals from caregiver counseling institutions take care of the caregiving situation and informal caregivers needs (focus on informal caregivers). The eHM-DP aims to provide access to relevant and comprehensive information for both professional groups in order to improve treatment and care and to prevent caregiver burden.

Before the final implementation and evaluation in a field trial, the eHM-DP was piloted in a pretest by 31 informal caregivers and 11 professionals [34]. Based on the pretest findings, the eHM-DP was revised. Major revisions were made on the following aspects: improved interaction functionality for communication between informal caregivers and professionals, the possibility for professionals to list patients based on priority levels (eg, worse health status), a revision of the medication plan, a print option for diaries and design aspects.

In the evaluation study, user access to the eHM-DP was realized via a customizable personal account for informal caregivers and professionals. Thus, the provision of individualized support services by means of a user-specific profile and user-specific diary entries (by informal caregivers) were enabled. This is important, as caregiver needs are multifaceted and complex in nature [16]. There are four major roles that are relevant for the eHM-DP:

1. Informal caregiver with own user account: provision of information about medical data and health status (symptoms, well-being) of the PwD (external assessment) as well as about their own caregiving situation and well-being (caregiver burden).
2. Person with dementia without personal eHM-DP account: PwD is not directly involved in the eHM-DP, but is indirectly via external assessment from informal caregivers and recommendations/advice from medical and social professionals with the aim to improve treatment, care, and well-being for the PwD.
3. Medical professional with personal eHM-DP account: acquisition of “hard-to-access” information about the PwD (improved decision making, improved treatment and care), timely reactions to health status changes of the PwD via the eHM-DP or directly.
4. Social professional with personal eHM-DP account: acquisition of “hard-to-access” information about the caregiving situation (prevention of caregiver burden), timely reactions to health status changes of the informal caregiver via the eHM-DP or directly.

**Figure 1:**
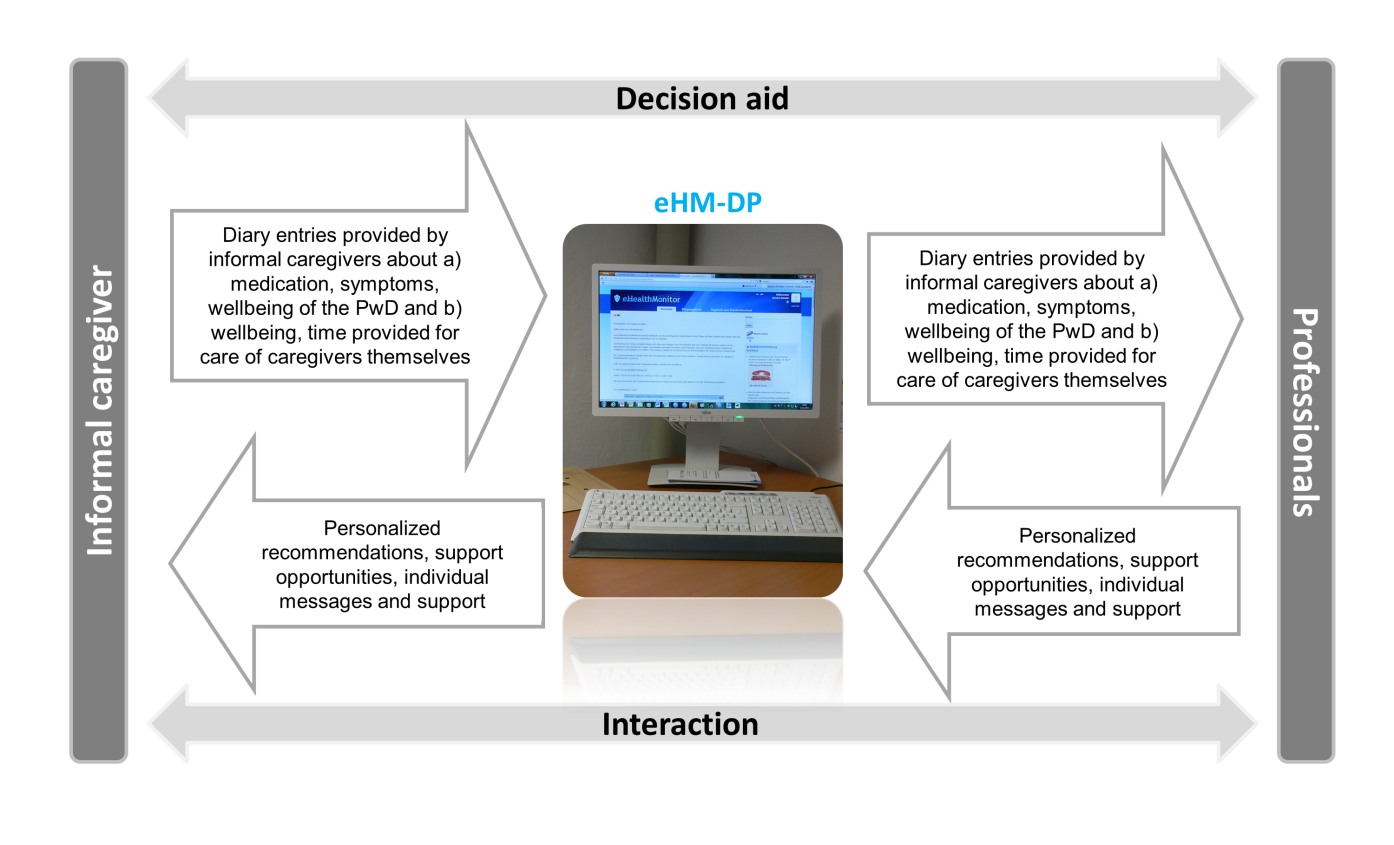
Schematic representation of the eHM-DP.

The eHM-DP is personalized and interactive and provides two major functionalities (see Figure 1): (1) interactive and individualized provision of information and knowledge, and (2) communication with domain experts in dementia. First, the portal provided individualized, timely, and situation-specific information to informal caregivers and professionals based on an individual registration profile as well as the electronic diary entries provided by informal caregivers (ie, caregiving diary, course-of-disease diary about PwD, medication diary about PwD). Informal caregivers received tailored information consisting of approved guidelines and documents (eg, factsheets of German Alzheimer Association, Ministry of Health, local dementia institutions/groups) and of recommendations from professionals via the message functionality of the eHM-DP. Second, the eHM-DP sought to facilitate and enable close communication and interaction between informal caregivers and professionals. Thus, its aim was to empower informal caregivers and prevent caregiver burden as well as to improve treatment and care for the PwD. From the perspective of professionals, the eHM-DP facilitated information acquisition of individual “hard-to-access” information and sought to improve the management, treatment, and care for PwDs (based on diary entries from caregivers about symptoms and medication of the PwD) and the well-being of informal caregivers (based on diary entries from caregivers about the caregiving situation and caregiver burden). Based on these individual diary entries provided by informal caregivers as well as specific questions (free text), professionals were informed by the eHM-DP (eg, alerts) and were able to provide support either via the portal (messaging feature) or directly (appointment, telephone call).

### Participants and Evaluation Design

The Consolidated Standards of Reporting Trials of Electronic and Mobile Health Applications and Online TeleHealth (CONSORT-EHEALTH) provided guidance for the development of our evaluation [35]. According to Eysenbach [35], these guidelines may also be used for other evaluation methods. Our evaluation study sought to assess the usefulness and impact of the final implementation of the eHM-DP in order to optimize the platform before starting a large-scale randomized controlled trial. This is in agreement with previous studies, which emphasized the need to conduct early evaluation studies such as “proof-of-principle-studies” before the realization of studies on a larger scale [36].

**Figure 2:**
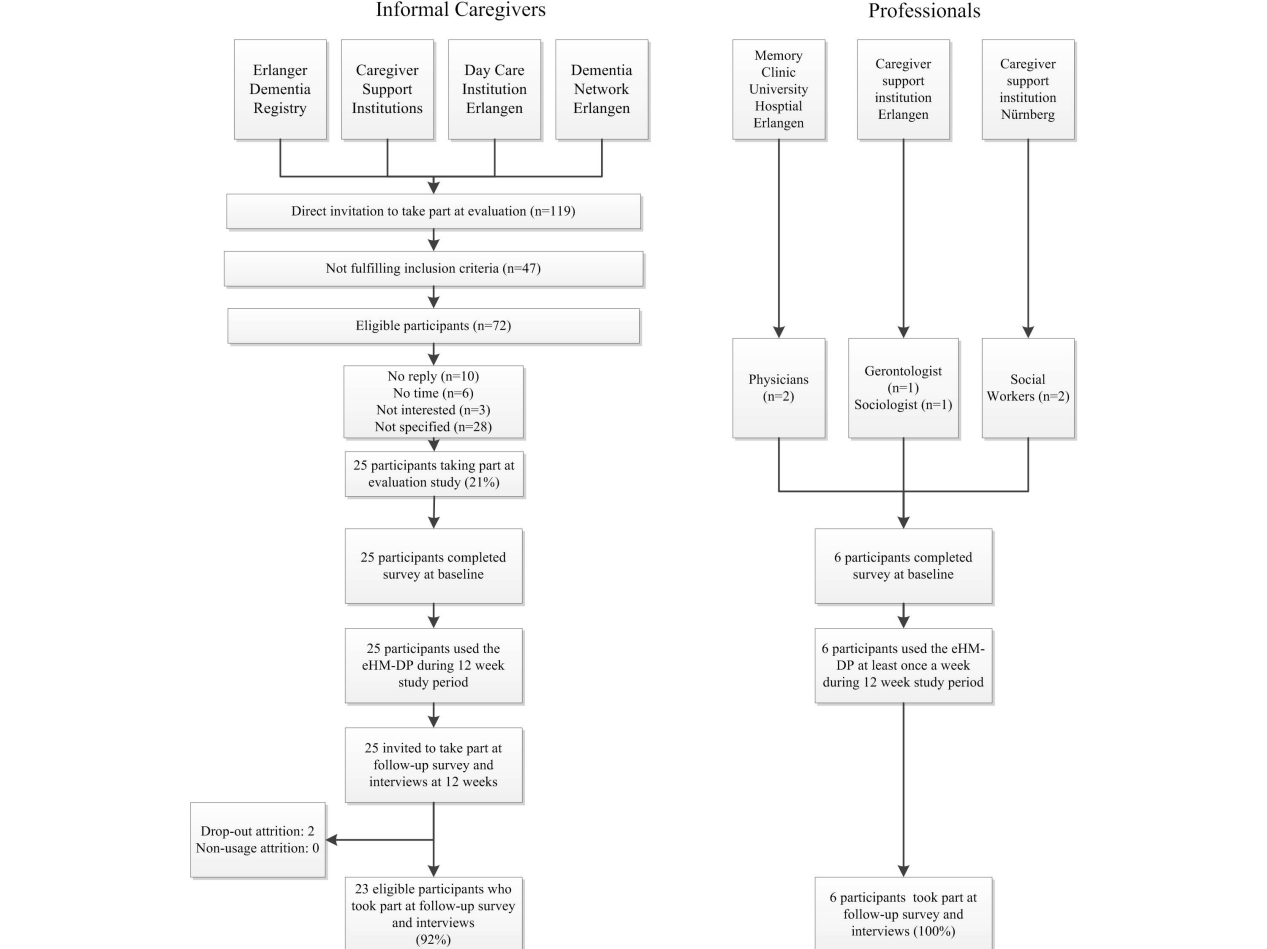
Schematic representation of the eHM-DP evaluation participant flowchart.

Our study was conducted from June 1-September 30, 2014, and followed a before-after study with 12-week follow-up to investigate the eHM-DPs impact on informal caregivers and professionals. Study participants accessed the Web portal from home (informal caregivers) or from their workplace (professionals) and used the Web portal at least once a week over the 12-week study period. A convenience sampling strategy was used to recruit study participants: informal caregivers, medical professionals, and social professionals. Informal caregivers were recruited from a Hospital Memory Clinic, a district hospital, and two caregiver support institutions from the metropolitan region of Erlangen-Nürnberg. Eligibility criteria included (1) primary responsibility as an informal caregiver for a person with dementia (International Statistical Classification of Diseases and Related Health Problems, ICD-10, F00-F03) who is living at home, (2) is aged over 18 years, (3) is able to speak, read, and write German, and (4) has Internet access from home. In addition, 2 medical professionals were recruited from a Hospital Memory Clinic and 4 social professionals from caregiver support institutions. The participant flowchart (see Figure 2) describes the recruitment and inclusion process in detail. The evaluation study was approved by the local ethics committee of the Friedrich-Alexander University Erlangen-Nürnberg (Germany). All study participants were informed about the objectives and the scope of the study and gave written, informed consent for participation. Data were collected confidentially in written face-to-face interviews by trained interviewers. Personal contact data (ie, name, address, institution) were used only for the second contact (follow-up interview) and related questionnaires were coded based on an identification number. The data analysis and interpretation were based on two separate datasets (informal caregivers and professionals) and conducted confidentially by 2 independent researchers.

### Procedure

The eHM-DP was introduced to study participants by trained persons according to the Standard Operating Procedure of eHealthMonitor’s evaluation [37]. The content of the introduction included a detailed explanation of all the features and functionalities of the eHM Portal and was supported with additional materials, such as videos and written information material. Support for study participants was provided throughout the whole study period (ie, telephone help desk, email). The non-usage attrition (discontinuation of services) [38] was minimized by “push” factors such as reminders and telephone calls.

### Measures

A user participatory research design based on a mixed-method approach was applied to investigate the different user perspectives of the eHM-DP at baseline and follow-up (after 12 weeks). This approach has been recommended in previous reviews and studies on eHealth interventions [21,39,40]. The questionnaire was developed according to our research questions and consisted of instruments assessing the perceived usefulness of the eHM-DP with regard to decision aid, the attitude toward using it, perceived benefits and concerns, as well as recommendations of both informal caregivers and professionals. In addition, the impact on informal caregivers’ quality of life and burden was explored.

Further, sociodemographic data as well as informal caregivers’ eHealth literacy, using the eHEALS scale [41], and needs, using the Carers’ Needs Assessment for Dementia (CNA-D) [30], were assessed at baseline.

#### Quantitative Measures

##### Usefulness: Empowerment (Informal Caregivers)

The empowerment of informal caregivers was assessed via 13 relevant categories of the CNA-D instrument and rated on a 5-point Likert scale (1=strongly agree to 5=not agree at all). Although the CNA-D was designed to assess carers’ needs, its relevant areas of need were chosen to assess whether the eHM-DP contributes to address these needs and is able to empower caregivers in these specific areas of need.

##### Usefulness: Decision Aid (Informal Caregivers, Professionals)

The “Preparation for Decision Making Scale” (PrepDM) [42] was used to assess the informal caregivers’ and professionals’ perception of the eHM-DP with regard to decision support. Based on 10 items, the preparation for decision making is rated on a 5-point Likert scale (5=a great deal, 1=not at all). The score ranges from 0 (no perceived preparation for decision making) to 100 (highest perceived level of preparation for decision making).

##### Usefulness: Attitude Toward Using (Informal Caregivers, Professionals)

The attitude toward using the eHM-DP was assessed via the item “I think that eHM is a good concept” and intention to use via the item‚ “If I had access, I would use eHM.” Both were rated on a 5-point Likert scale (1=strongly agree to 5=not agree at all). In addition, users were asked about the frequency of use of the eHM-DP.

##### Usefulness: Perceived Benefits (Informal Caregivers, Professionals)

The benefits for each user group were assessed via specific items that were rated on a 5-point Likert scale (1=strongly agree to 5=not agree at all). The questionnaire items were derived from current literature as well as pretest results of the eHM-DP [34].

##### Impact: Quality Of Life (Informal Caregivers)

Health-related quality of life was measured via the EQ-5D-5L instrument [43], which captures five dimensions: mobility, self-care, activity, pain, and anxiety. The labels for each of the dimensions are no problems, slight problems, moderate problems, severe problems, and unable to/extreme problems. Utility values range from 1 (best possible health) to 0 (worst possible health).

##### Impact: Caregiver Burden (Informal Caregivers)

Caregiver burden was assessed via the short form of the Burden Scale for Family Caregivers (BSFC) [44], which measures the subjective caregiver burden of informal caregivers. It consists of 10 items rated on a scale (3=strongly agree, 2=agree, 1=disagree to 0=strongly disagree). The score ranges from 0-30 (0-9: low burden; 10-20: moderate burden; 21-30: severe burden).

#### Qualitative Interviews

In addition to the quantitative data, the perceived usefulness was explored through a semistructured interview focusing on users’ experiences in terms of perceived benefits, major concerns, and further desired functionalities and improvements. The rationale was to assess the impact on users’ perception of the eHM-DP. The semistructured, written interviews lasted approximately 60 minutes. All interviews were performed by a trained interviewer (3 interviewers were involved in the study) at baseline and follow-up. Interviews were conducted either at the study participants’ home (informal caregivers) or at work (professionals).

### Data Analysis

Descriptive analysis methods for quantitative data were applied using SPSS Statistics 21.0 software. Paired sample *t* tests were used to analyze changes in caregiver burden and quality of life from baseline to 12 weeks. Written qualitative data was captured electronically and structured by the method of an inductive category development according to Mayring [45]. The summary content analysis technique was applied in order to reduce the material to core contents or aspects. Therefore, the following steps were applied: (1) paraphrasing of content-bearing text passages, (2) generalization to the required level of abstraction (category definition), (3) first reduction through selection, and (4) second reduction [45]. At the end of this reduction phase, exact checking took place to ascertain whether the new statements collated as a category system still represent the base material. An intercoder check by 2 coders was performed to assure quality of the data analysis. The software MAXQDA was used to conduct the content analysis.

## Results

### User Statistics

A total of 119 informal caregivers were invited to take part in the evaluation study. Of those, 47 (39.5%) did not meet the inclusion criteria (eg, no Internet access), and 25 (35%) of the 72 caregivers who were qualified for the study were interested in taking part in the study. The main reason for non-participation was “no time,” which often was due to the fact that informal caregivers were already stressed by the caregiving situation. This fact has to be taken into account when interpreting our conclusions and when planning further studies in the field. Informal caregivers were aged 29-80 years. Mean age was 58 years (SD 13.0, median 61 years) and almost half (48%, 12/25) of informal caregivers were female. Informal caregivers were mainly spouses (44%, 11/25) or children (36%, 9/25) of the PwD. Table 1 provides detailed information for the 25 informal caregivers.

**Table 1:** Informal caregivers’ characteristics (N=25).

		Participants, n (%)	Median; Min, Max
**Age**			Median 61; min=29, max=80
Time caring for PwD (years)			4 (3); min=1, max=12
**Sex**			
	Female	12 (48)	
	Male	13 (52)	
**Relationship to PwD**			
	Spouse	11 (44)	
	Child	9 (36)	
	Relative	5 (20)	
**Living situation**			
	Living together with PwD	14 (56)	
	Living NOT together with PwD	11 (44)	
**Living area**			
	Urban	16 (64)	
	Rural	9 (36)	
**Professional status**			
	Full-time employed	9 (36)	
	Part-time employed	4 (16)	
	Retired	10 (40)	
	Other	2 (8)	
**Caregiver burden**			
	Low	11 (44)	
	Moderate	8 (32)	
	Severe	6 (24)	

**Figure 3:**
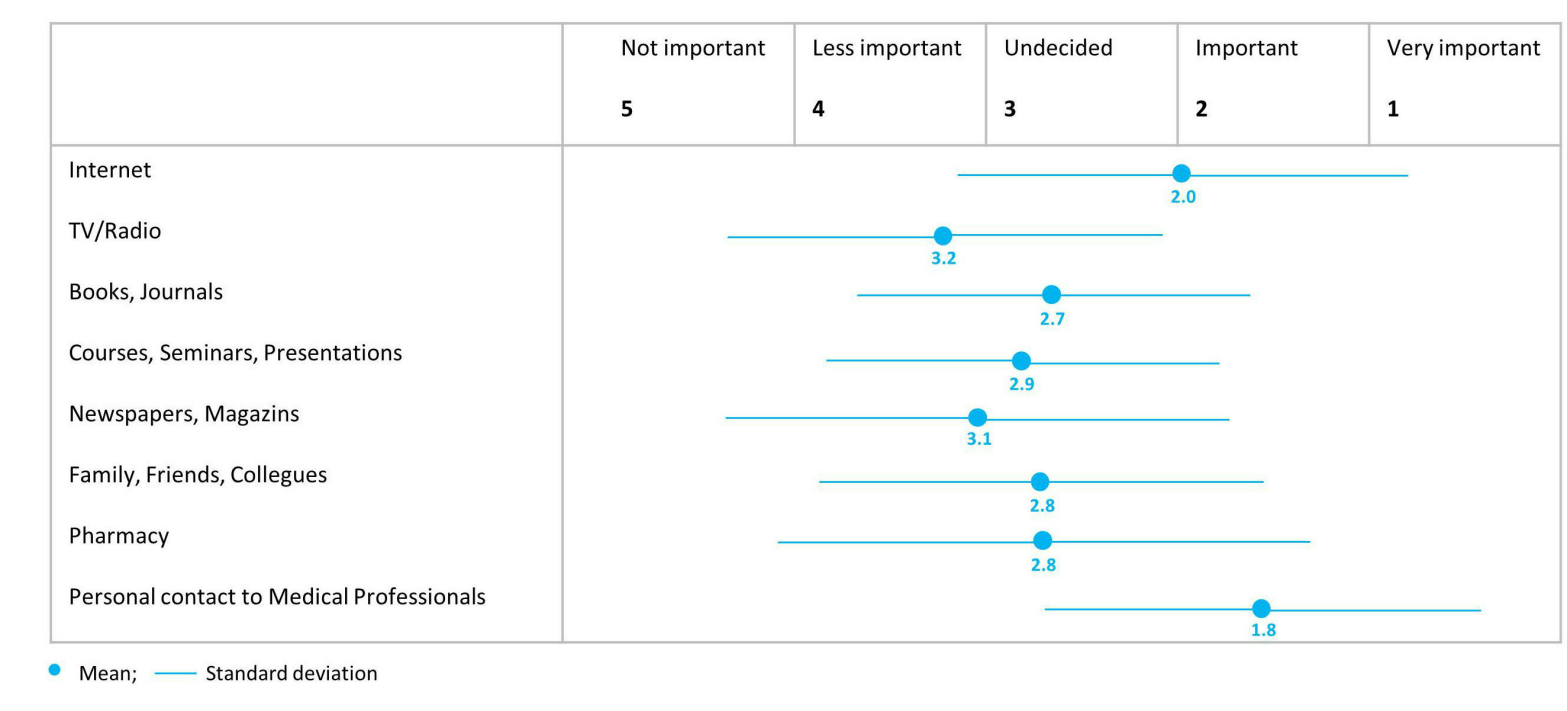
Importance of information sources for health-related aspects.

At baseline, the health information source “Internet” was rated as “very important” by 48% (12/25) and as “important” by 24% (6/25) of informal caregivers. It is rated as the second most important information source (mean 2.0, SD 1.2), directly after the personal contact to professionals (mean 1.8, SD 1.1) (see Figure 3). eHealth literacy competence at baseline was measured by the eHEALS Scale [41] and indicated a mean value of 19.9 (SD 9.0) on a scale from 8-40. On average, 37% of informal caregivers expressed needs for one of the 18 categories of the CNA-D instrument. The mean value for the number of reported needs is 6.6 (SD 5.3).

Mean age of professionals was 43 years (SD 13.2; min=31; max=58) and half of them were female (3/6). In total, 29 study participants (23 informal caregivers and 6 professionals) took part in the follow-up study (two drop-out attritions within the group of informal caregivers; reasons: 1 PwD died, 1 informal caregiver was sent to hospital) [38].

###  Quantitative Findings

#### Usefulness: Empowerment (Informal Caregivers)

**Figure 4:**
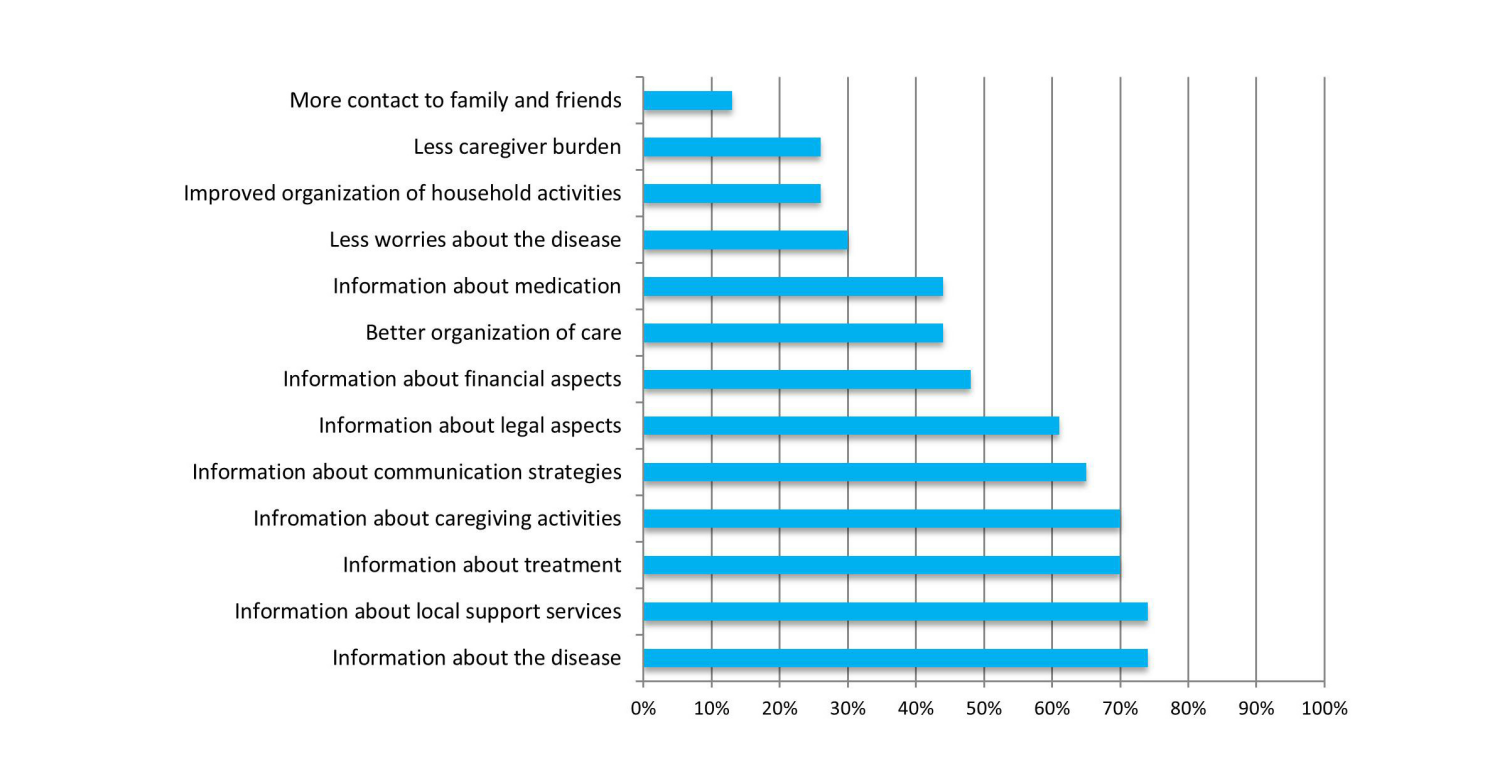
Empowerment via the eHM-DP (Informal caregivers, n=23).

The empowerment of informal caregivers was assessed via 13 relevant categories of the CNA-D instrument (see Figure 4). The most perceived empowerment was received via information acquisition for several topics (disease, local services, treatment, caregiving activities, and communication strategies). About a third agreed that the eHM-DP contributed to fewer worries about the disease or to less caregiver burden.

#### Usefulness: Decision Aid (Informal Caregivers, Professionals)

**Figure 5:**
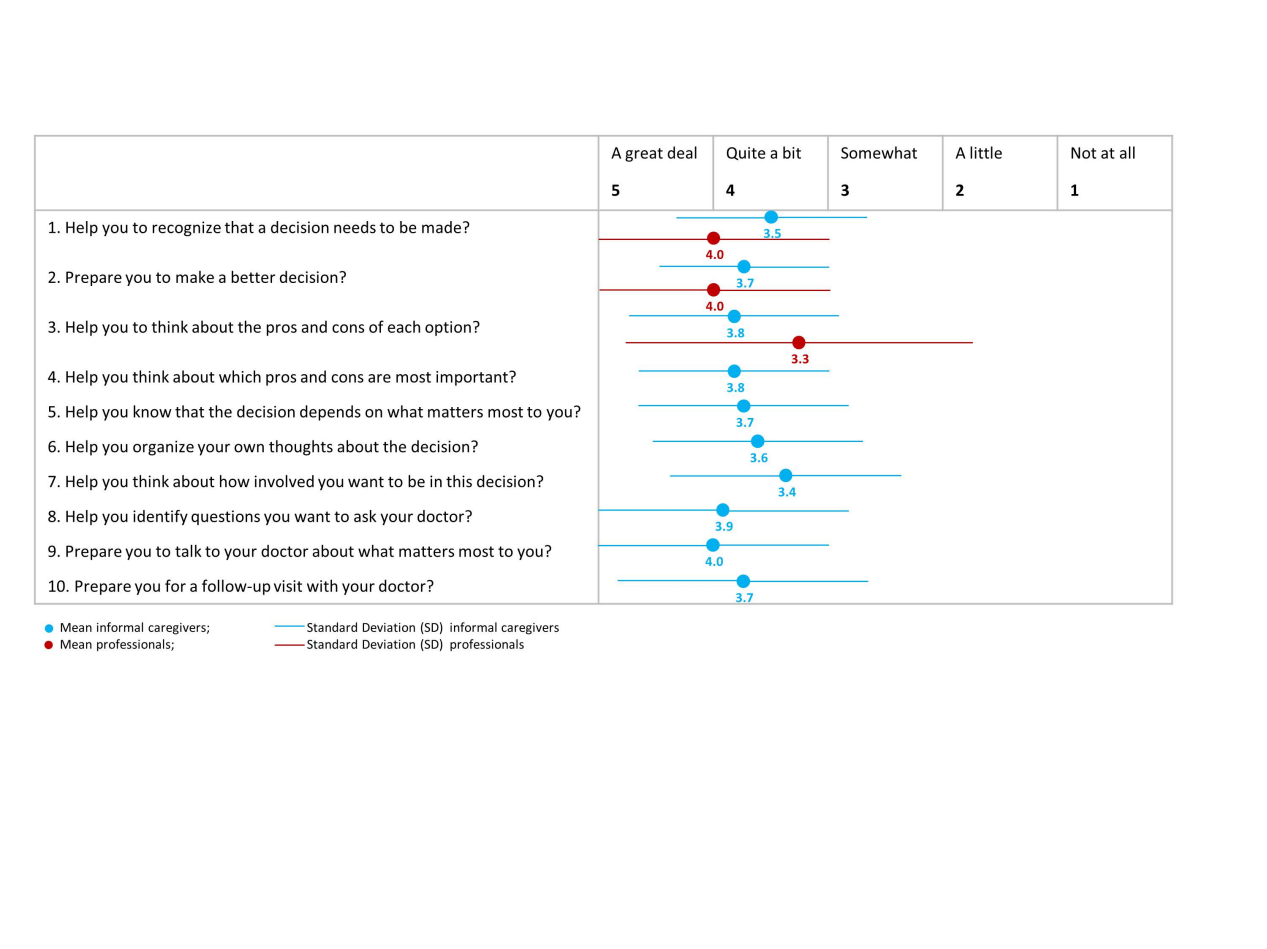
Preparation for decision making by the eHM-DP service.

The score of 67.9 (min=38.9; max=100.0) of the PrepDM instrument indicates the perceived decision aid of the eHM-DP for informal caregivers. Highest decision support was perceived for “Prepares me to talk to my doctor about what matters most to me” (mean 4.0, SD 1.0), followed by “Helps me to identify questions I want to ask my doctor” (mean 3.9, SD 1.1). Professionals pointed out that eHM-DP specifically helps them to recognize that a decision has to be made (mean 4.0, SD 1.0) and prepares them to make a better decision (mean 4.0, SD 1.0). Figure 5 illustrates the perceived decision support of the eHM-DP.

#### Usefulness: Attitude Toward Using (Informal Caregivers, Professionals)

Two thirds (65%, 15/25) of informal caregivers indicated that they would use the eHM-DP service if they had access to it. In total, 83% (19/25) of caregivers and 67% (4/6; 2 medical professionals, 2 social professionals) of professionals think that eHM-DP is a good concept. Further, 2 social professionals were not convinced that eHM-DP is a good concept; however, they suggested further functionalities that would make the eHM-DP unique for them (eg, involvement and connection of all relevant stakeholders within the dementia treatment and care process via the eHM-DP). With regard to the informal caregivers’ frequency of use, 22% (5/25) indicated 2-3 times per week, 57% (13/25) weekly, and 13% (3/15) monthly. Half of the professionals prefer to use the eHM-DP at least once a week.

#### Usefulness: Perceived Usefulness (Informal Caregivers, Professionals)

**Figure 6:**
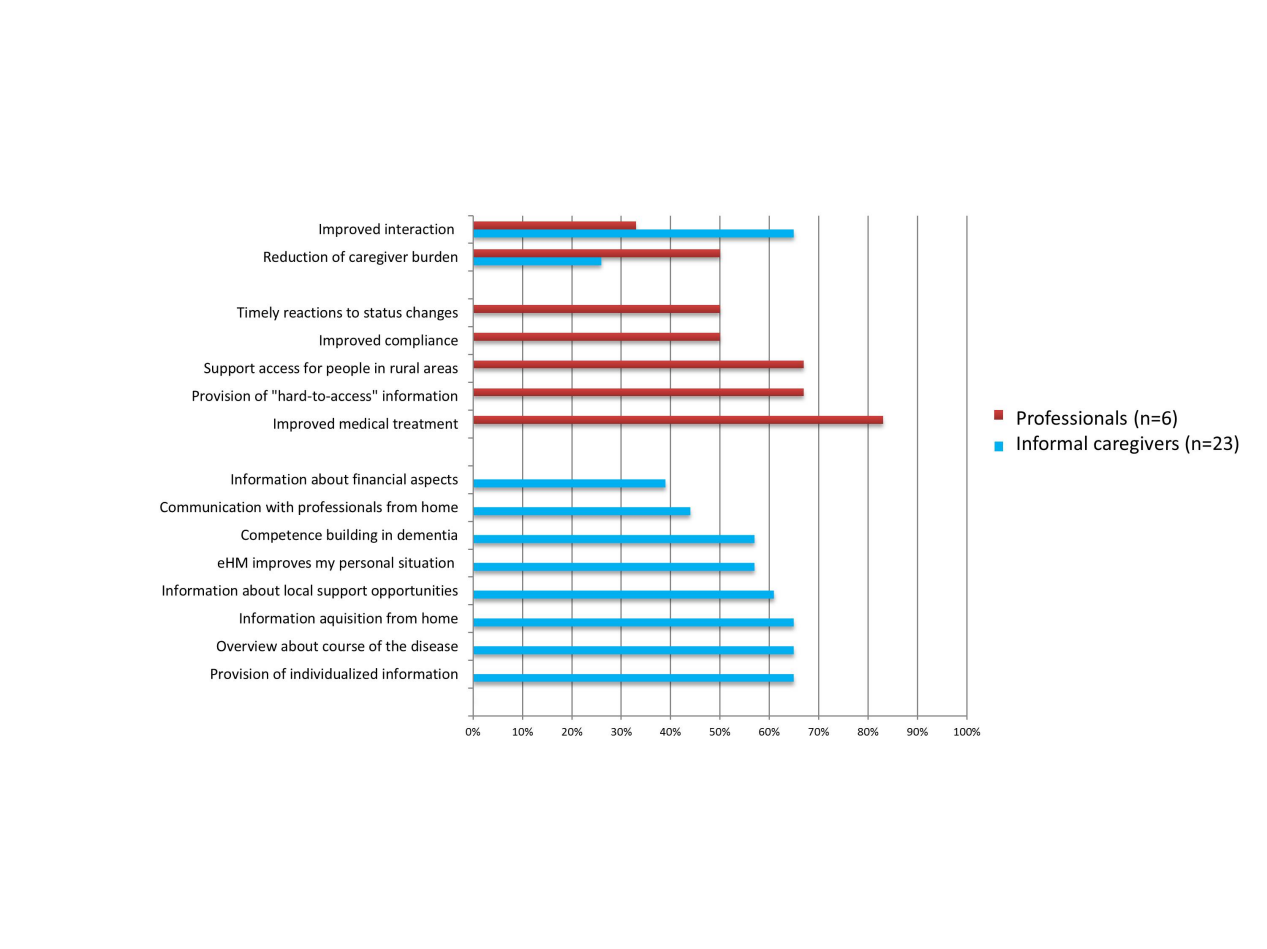
Perceived usefulness of the eHM-DP.

Although there were no pre-post changes in quality of life, the user-specific questionnaires suggest that over half of the informal caregivers perceived an improvement of their individual situation (see Figure 6). Over one third highlighted an improvement in communication with professionals (especially from home), the provision of individualized information, an overview about course of the disease (symptoms, care), as well as the information acquisition from home. In addition, the provision of information about local support opportunities, the improved individual situation as an informal caregiver and the competence-building in dementia were mentioned. A resulting consequence for 26% (6/25) of informal caregivers is a reduction of caregiver burden. From the perspective of professionals, the eHM-DP contributes to an improved medical treatment of the PwD (83%, 5/6) and improved compliance (50%, 3/6). One major aspect that contributed was the provision of “hard-to-access” information (home-based care setting, symptoms over disease course). This resulted in faster response time to status changes and thus also to a reduction of caregiver burden (50%, 3/6), and overall improved interaction with informal caregivers (33%, 2/6). A third aspect that is highlighted is the improved access to support, especially for mobility-impaired persons and for people living in rural areas (67%, 4/6). In addition, professionals indicated that eHM-DP contributes to active medical decision making for PwD and informal caregivers, timely reaction to changes in the disease course (67%, 4/6), as well as the prevention of the deterioration of the disease (50%, 3/6).

Overall, medical professionals emphasized the possibility of timely reactions to status changes, whereas social professionals highlighted the improved access to care and support for caregivers who are living in rural areas. Both groups showed a high level of consistency with regard to an improved medical treatment of the PwD.

#### Impact: Quality of Life and Caregiver Burden (Informal Caregivers)

After 12 weeks of eHM-DP usage, there were no improvements on primary informal caregiver outcomes (BSFC and EQ-5D-5L).

### Qualitative Findings

**Figure 7:**
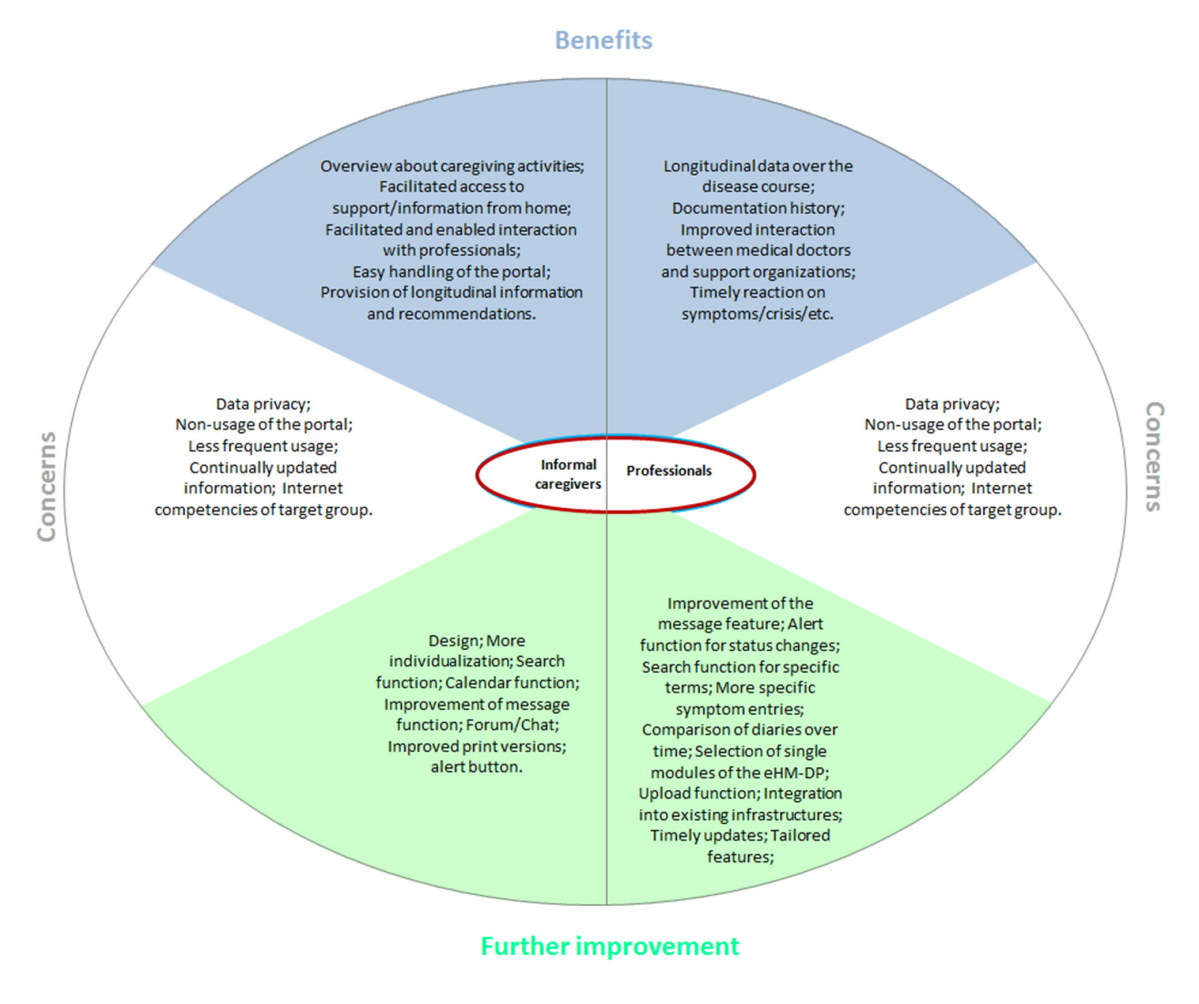
Results of qualitative content analysis: Benefits, concerns, improvements.

The content analysis of the 29 interviews (23 informal caregivers and 6 professionals) resulted in 18 categories across three domains (ie, perceived benefits, concerns, further improvement), which are presented below (see Figure 7).

#### Perceived Benefits (8 Categories)

Informal caregiver reported the following five major benefits: saved time, 24-hour access, facilitated communication with professionals, easily operated portal, and overview about individual caregiving activities.

A major benefit for informal caregivers was saved time (eg, communication with professionals from home, provision of individualized information), which was predominantly expressed by employed informal caregivers. Another perceived benefit was 24-hour facilitated access to support and information, due to access from home or work. A further perceived benefit resulted from the facilitated and increased communication with professionals. In addition, the overview about individual caregiving activities resulted in increased awareness of personal tasks and support areas. The easily operated portal also accounted for frequent use from users.

For the professionals, the following four major benefits were identified: provision of longitudinal data over the disease course, documentation history, improved interaction between professional institutions and support organizations, and timely reaction to status changes.

Provision of longitudinal data over the disease course and documentation history were essential and beneficial factors of the eHM-DP. In addition, the professionals mentioned an improved interaction between professional institutions and support organizations. The latter factors contributed also to an improved and faster response time to behavioral symptoms, adverse events, or crisis.

#### Concerns (4 Categories)

We identified the following categories: concerns of data privacy, risk of non-usage of the portal, assurance of provision of up-to-date information, and insufficient Internet competence of informal caregivers. Both informal caregivers and professionals expressed concerns about data privacy (eg, who will access the entered data?). In addition, they mentioned the risk of non-usage or insufficiently frequent usage, which is the precondition to realize the eHM-DP’s benefits and interaction between both sides. Further, they emphasized that all provided information has to be up to date and professionals stated that the target group of informal caregivers is hard to reach and often has less competence with the Internet.

#### Further Improvement (6 Categories)

The following categories for the domain “further improvement” were identified: improved design of the portal, more specific individualization of the portal, further functionalities, improvement/extension of specific functionalities, selection of certain functionalities of the portal for professionals, and integration of the eHM-DP into existing infrastructures.

Informal caregivers reported the following comments for further improvements. First, they suggested an improved design of the eHM-DP (eg, the presentation of tables and graphs, the main menu, the selective use of colors, or the presentation of videos). In addition, they emphasized the need for even more specific individualization of the eHM-DP (eg, according to the caregiver status to the patient [spouse or child] or for specific topics). The possibility of searching for specific terms, information about local events (eg, based on a calendar), print version for whole diaries, and the integration of an alert button were warranted. Further, the need to improve the message feature for communication with professionals was expressed by both informal caregivers and professionals (eg, display of a message history, read confirmation). In addition to the interaction with professionals, informal caregivers wanted to have an integration of a forum or chat for informal caregivers themselves.

The professionals mentioned the benefit of an improved alert function for status changes of the patient or caregiver, which helps them react in a timely fashion to status changes. In addition, the possibility of making more specific symptom entries and caregiving entries within the diaries and the integration of a search functionality for specific terms was expressed. Professionals would also benefit from the option to compare diary entries (over time). A selection of single modules of the eHM-DP service would help professionals choose the most important functionalities of the service and tailor the eHM-DP features and modules to specific professions (eg, physician, caregiver counseling institution, psychologists, care service). Also the possibility of uploading specific documents like blood parameters or diagnostic findings would facilitate patient management.

Finally, the need for integration of the eHM-DP into existing software infrastructures as well as the need for timely updates of provided information was highly warranted.

## Discussion

### Principal Findings

This study describes the perceived usefulness and impact of a personalized eHealth service (eHM-DP) that aims to support informal caregivers and professionals in the dementia treatment and care setting. Our results reveal that the eHM-DP is promising in addressing needs of both informal caregivers [2,3,30,31,33] and professionals [32] in the dementia treatment and care setting. Those were addressed by five major factors: individualized information acquisition, decision support, facilitated access to care and support infrastructures, interaction between professionals and informal caregivers, and provision of longitudinal data about the disease course and medication history (“hard-to-access” information). Although pre-post changes in caregiver burden (BFSC) and quality of life (EQ-5D-5L) for informal caregivers were not observed, the perceived usefulness of the eHM-DP and qualitative interview data of both study participant groups are encouraging. They indicated perceived benefits and a positive impact of the eHM-DP with respect to increased knowledge in certain topics (PrepDM, CNA-D), as well as the facilitated interaction with professionals. These results are also evident in a recent review from Boots et al (2014) [22], concluding that multicomponent interventions that combine tailored information with interaction are the most promising approaches [22].

One factor that contributed to these results is that both user groups have been involved early in the development process of the eHM-DP, which has proven to be valuable in previous studies [46-48]. Further, the combination of comprehensive information provision within the eHM-DP of informal caregivers and of PwDs was relevant for tailoring information and support.

From the perspective of professionals, the eHM-DP provided benefits for improved medical treatment of dementia patients, improved compliance, and interaction with informal caregivers. Although no significant changes in the reduction of caregiver burden were measured, half of the professionals considered that the eHM-DP is able to reduce caregiver burden. The latter aspects are encouraging with regard to the reduction of hospitalizations and institutionalizations (positive health economic impact), as especially caregiver burden is one of the main predictors for nursing home referrals [9,10,49]. A major factor that led to these perceived benefits was the timely provision of “hard-to-access” information (eg, home-based care situation, symptoms over disease course, medication, subjective informal caregiver burden, health status of informal caregiver and PwD). This is a major advantage as informal caregivers are often the only information source for professionals at (regular) checkups but are not able to provide quantified long-term details. However, professionals expressed doubts about whether the informal caregivers would be motivated to use the eHM-DP regularly. This concern could not be confirmed within our study; however, participants were reminded and motivated to use the eHM-DP. The physicians from the memory clinic mainly benefited from the course of disease diary with timely, quantified, and longitudinal information about cognitive status, activities of daily living, mood, behavioral symptoms, and social behavior, followed by the medication diary with information of all medications and reported side effects. This is especially important because PwDs are often elderly persons with more than one disease who are in contact with several physicians [50]. The latter diaries enable an overview of all prescriptions, side effects, and comorbidities of the PwD. In contrast to physicians, caregiver counseling institutions emphasized the comprehensive (PwD and informal caregiver status, needs) and timely information provision that helps prevent caregiver burden and provides additional benefits (eg, optimized counseling, improved accessibility for target group) to the usual face-to-face support. The eHM-DP provided them additional and timely insight into actual carers’ needs and, thus, contributes to prevention of caregiver burden and early crisis intervention. This is crucial, as informal caregivers often wait until a crisis situation occurred or they become exhausted before seeking support [51]. Regular contact with professionals can help prevent such crisis situations.

From the perspective of informal caregivers, the provision of individualized information for the specific care situation of the informal caregiver contributed to caregiver empowerment. The importance of tailoring information to informal caregivers’ needs was highlighted in previous studies [21,23,33,52,53]. Within the eHM-DP, modifying information based on the symptoms and status of the PwD, in addition to informal caregivers’ health status and needs, proved to be important for individual information provision and interaction with professionals. In addition to provided information from the eHM-DP, professionals gave individual recommendations based on diary entries via the portal message feature. A number of the informal caregivers reported that information about specific topics triggered reflection about their own caregiver role and situation and, thus, provided a useful decision aid. In contrast to experienced caregivers, “new” caregivers of recently diagnosed PwDs reported a high perceived benefit with regard to individual information acquisition.

Overall, a recent review indicated that informal caregivers often receive little or unclear information about dementia, especially after the diagnosis disclosure [54]. This leads to another determinant that contributed to empowerment of informal caregivers: the facilitated access to information and support. It is evident that the accessibility of services is a major predictor for its utilization [11,55]. The overall empowerment of informal caregivers via knowledge acquisition about local support options is crucial, as the lack of knowledge about existing services and dementia infrastructures contributes to one of four major reasons for non-use of services [10]. The eHM-DP contributes to increased autonomy in the sense of awareness and offers the chance to choose between different support services as demonstrated by Schüz et al [56]. In particular, informal caregivers from rural areas, non-mobile informal caregivers or full-time employed informal caregivers emphasized the facilitated interaction and information acquisition from home. In addition, it can reach informal caregivers, who are either not motivated (eg, persons who do not want to travel to the next town) or not able to use (eg, no public transport) traditional services like caregiver counseling. This is especially true for people living in rural and remote areas [18,51,57]. These findings are in line with previous studies, where time constraints, transportation, and health issues have been identified as predictors for non-participation in face-to-face support services [12,51].

### Limitations

Although the findings of our study provided essential insight into the usefulness of eHealth support services for informal caregivers of PwD and professionals, some limitations need to be considered. Our study confirmed that introducing eHealth services to elderly people is challenging, as computer competencies and Internet access are important prerequisites, which narrows the potential number of study participants. The objective was to conduct a comprehensive proof of principle study with a demonstrator in order to improve the eHM-DP before evaluating effects on a larger scale and in a (randomized) controlled group design. This approach has been recommended in previous studies [36]. However, the fact that no control group was included is a limitation and the realization of a controlled study is needed. The first limitation was the small sample size of study participants. The convenience sample had rather lower levels of caregiver burden, which has to be taken into account when interpreting results for further studies on a larger scale, as well as the reach of the intervention. Additionally, the positive feedback of the eHM-DP from 83% of caregivers may be overestimated due to the convenience sample that agreed to take part in the study.

Another limitation is the short 12-week study period, which likely explains the non-significant changes in quality of life and caregiver burden. Furthermore, an apparent discrepancy between the non-significant pre-post outcome measures (BSFC, EQ-5D-5L) and the positive results on perceived usefulness of both user groups is present. This can be explained by the small number of study participants and the testing period of 12 weeks. However, an advantage of our study is the focus on post-outcome measurements and qualitative interviews (mixed-method design) that provided promising results for the eHM-DP service and emphasizes the need for further controlled studies on a larger scale.

### Future Directions

Our study contributes to scientific research by providing insight into the different user perspectives of eHealth support services in the dementia treatment and care setting. These results are crucial for future developments and the use of eHealth solutions in the dementia domain and reinforce the importance of early user involvement. The perceived benefits and willingness to use the system combined with an increasing number of adults who use the Internet regularly, emphasizes the potential of personalized and Web-based support services for informal caregivers. This is especially true for our aging societies and limited expenditures for health care services.

Based on our findings, the following aspects are decisive for implementing eHealth services in the dementia treatment and care context: a comprehensive introduction into the eHM-DP with all its functionalities, the provision of informational material and the provision of a contact person for seeking advice or help. These aspects proved to be important to ensure competent handling of the eHM-DP service. In addition, the latter aspects are suitable for addressing concerns of both study participants (non-usage of the portal, Internet competencies of target group, data privacy). Furthermore, the provision of technical support is essential, in particular for participants with low computer competence. In the future, the introduction and support could be provided by, for example, the professional organization itself or by trained, voluntary caregivers. The use of reminders (eg, messages, phone calls) to use the eHM-DP has proven to be valuable.

Another important success factor of the eHM-DP is the facilitated and enabled computerized interaction between informal caregivers and professionals. Future developments of the eHM-DP should concentrate on linking more than one stakeholder via the eHM-DP (eg, informal caregiver, social worker, physician, professional caregiver) because a well-functioning, interprofessional, and interorganizational communication in dementia care is important [58]. Overall, further controlled studies on a larger scale, focusing on cost-effectiveness and usability are crucial to embed the eHM-DP into existing health care infrastructures. In addition, the future eHM-DP development should exploit potential synergy effects between existing systems with similar intentions, such as InformCare [59] as well as complementary systems such as Ambient Assisted Living (AAL)-systems (eg, ALLADIN [60]), educational online courses (eg, Mastery over Dementia [61]), or chatrooms (eg, ALZConnected [62], ANKER [63]).

In particular, different priorities and needs of professional organizations (eg, hospitals versus counseling institutions or professional caregivers) have to be considered carefully. Valuable tips for professional organizations were already provided in the results section. In addition, more research with regard to different informal caregiver types (eg, according to gender, relationship to PwD, employment status, rural areas, and ethnicity) and related needs is necessary. This was also emphasized in the qualitative study results, where informal caregivers expressed the wish for more individualization.

Turning to the primary target of the eHM-DP service, our findings suggest that the eHM-DP service proved to be a valuable post-diagnostic eHealth support service for the home-based care setting. It revealed several benefits for families (informal caregivers), professionals, and health care systems, which are the basis for further studies and future health care policy planning in dementia.

## Abbreviations

BSFC: Burden Scale for Family Caregivers
CNA-D: Carers’ Needs Assessment for Dementia
eHM: eHealthMonitor
eHM-DP: eHealthMonitor Dementia Portal
ICD: International Statistical Classification of Diseases and Related Health Problems
MAS: multi-agent system
PeKS: Personal eHealth Knowledge Space
PrepDM: Preparation for Decision Making Scale
PwD: person with dementia
